# Epigenetics of hypoxic pulmonary arterial hypertension following intrauterine growth retardation rat: epigenetics in PAH following IUGR

**DOI:** 10.1186/1465-9921-14-20

**Published:** 2013-02-14

**Authors:** Xue-Feng Xu, Ying Lv, Wei-Zhong Gu, Li-Li Tang, Jia-Kai Wei, Li-Yan Zhang, Li-Zhong Du

**Affiliations:** 1 Department of Respiratory Medicine, the Children's Hospital, Zhejiang University School of Medicine, Hangzhou, 310003, P.R. China; 2 Department of Neonatology, the Children's Hospital, Zhejiang University School of Medicine, Hangzhou, 310003, P.R. China; 3 Department of Pathology, the Children's Hospital, Zhejiang University School of Medicine, Hangzhou, 310003, P.R. China

**Keywords:** Epigenetics, Pulmonary arterial hypertension, Endothelial cells, Endothelin-1, Intrauterine growth retardation

## Abstract

**Background:**

Accumulating evidence reveals that intrauterine growth retardation (IUGR) can cause varying degrees of pulmonary arterial hypertension (PAH) later in life. Moreover, epigenetics plays an important role in the fetal origin of adult disease. The goal of this study was to investigate the role of epigenetics in the development of PAH following IUGR.

**Methods:**

The IUGR rats were established by maternal undernutrition during pregnancy. Pulmonary vascular endothelial cells (PVEC) were isolated from the rat lungs by magnetic-activated cell sorting (MACS). We investigated epigenetic regulation of the endothelin-1 (ET-1) gene in PVEC of 1-day and 6-week IUGR rats, and response of IUGR rats to hypoxia.

**Results:**

The maternal nutrient restriction increased the histone acetylation and hypoxia inducible factor-1α (HIF-1α) binding levels in the ET-1 gene promoter of PVEC in IUGR newborn rats, and continued up to 6 weeks after birth. These epigenetic changes could result in an IUGR rat being highly sensitive to hypoxia later in life, causing more significant PAH or pulmonary vascular remodeling.

**Conclusions:**

These findings suggest that epigenetics is closely associated with the development of hypoxic PAH following IUGR, further providing a new insight for improved prevention and treatment of IUGR-related PAH.

## Introduction

An adverse intrauterine environment, such as uteroplacental vascular insufficiency and maternal malnutrition, may impact the development of the fetus resulting in fetal growth restriction or intrauterine growth retardation (IUGR) [1,2]. Their perinatal mortality is four to ten times higher than that of normally grown babies [1,3]. Moreover, a large number of epidemiological studies reveal that IUGR or low birth weight has been linked to the later development of diseases in adulthood, including type 2 diabetes and hypertension [4-6].

Compared with large for gestational age (LGA) infants, IUGR or small for gestational age (SGA) infants show a significantly greater risk of developing chronic lung disease (CLD) [7]. Premature infants with extremely low birth weight who presented initially with no lung disease or only a mild form of respiratory distress syndrome visible on chest radiograms may have an acute pulmonary hypertension episode and right ventricular dysfunction [8]. Moreover, a transient perinatal insult to the pulmonary circulation could have a persistent effects that, when activated later in life, predisposes to developing significant pulmonary vasoconstriction. These survivors would be at a greater risk of developing pulmonary arterial hypertension (PAH) later in life [9]. Another study demonstrated that hypoxia-induced IUGR rats had important deleterious consequences on the cardiopulmonary function of the offspring later in life, resulting in left ventricular diastolic dysfunction and PAH [10]. Accumulating evidence revealed that IUGR induced by intrauterine adverse environment can cause varying degrees of PAH or pulmonary vascular remodeling.

There is evidence that epigenetics play an important role in the fetal origin of adult disease [11-14]. Epigenetics refers to all heritable changes in phenotype or in gene expression states that are not involved in the DNA sequence itself, including histone modification and DNA methylation [15]. To date, epigenetic regulations in adult onset diseases following IUGR, including type 2 diabetes and hypertension, have been extensively investigated [5,15-17]. Rexhaj et al. found that in mice maternal undernutrition would induce pulmonary vascular dysfunction in the offspring by a DNA methylation of lung tissue [18]. However, data about whether epigenetic regulation is involved in the pathogenesis of PAH following intrauterine environmental changes is limited.

In a previous study, we found that the epigenetic modification of the endothelial nitric oxide synthase (eNOS) gene in persistent pulmonary hypertension of the newborn (PPHN) rats induced eNOS protein expression [19]. However, the increased eNOS expression did not completely offset the effect of vasoconstrictors, such as endothelin-1 (ET-1), and subsequent development of PPHN [19,20]. A number of clinical studies demonstrated that increased ET-1 was strongly associated with PAH [21-23]. Similar to the eNOS gene, the ET-1 gene expression was also regulated by epigenetics [24-26]. Some transcription factor binding sites, including hypoxia-inducible factor-1α (HIF-1α), are present in the ET-1 proximal promoter region, of which, HIF-1α is a key transcription factor in the hypoxia-induced ET-1 gene expression [27,28].

Based on the theory that epigenetics plays an important role in the developmental origins of adult disease, we hypothesized that IUGR induced by nutrient restriction could cause epigenetic modification of the PAH-related genes (ET-1), that persists into later life. These epigenetic changes would induce individuals to be highly sensitive to hypoxia or other stimuli, resulting in PAH or pulmonary vascular remodeling. To better understand the role of epigenetics, we performed this study at a cellular level by magnetic-activated cell sorting (MACS).

## Methods

### Intrauterine growth retardation (IUGR) rat model

**Figure 1:**
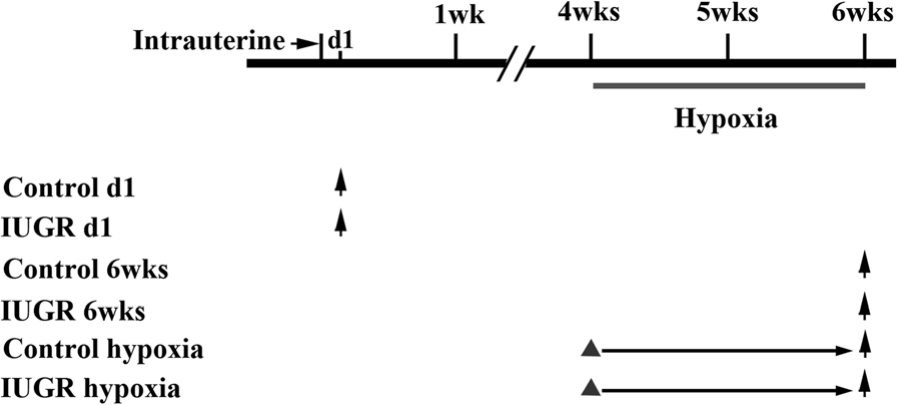
**The sampling age of different experimental group rats.** Up arrow represents sampling time, solid triangle represents the time of intervention, d1 represents 1 day after birth.

This study was carried out in strict accordance with the recommendations in the Guide for the Care and Use of Laboratory Animals of the National Institutes of Health. All procedures and protocols were approved by the Committee on the Ethics of Animal Experiments of Zhejiang University. All surgery was performed under anesthesia, and all efforts were made to minimize suffering. The IUGR rat model was established based on our previous study [29]. Virgin female Sprague–Dawley rats weighing 250-300 g obtained from Zhejiang University Laboratory Animal Center were mated overnight. After confirmation of mating, the pregnant rats were randomly divided into two nutritional groups: standard diet ad libitum throughout gestation (Control group), and undernutrition group. Pregnant rats in the control group were fed a standard commercial rat diet, while pregnant rats in the undernutrition group were fed the same diet at 50% of the free intake until birth. Both groups of rats were kept in the same room with a constant temperature maintained at 22 ± 3°C, and free to drink. Food intake and maternal weights were recorded daily until delivery. The establishment of IUGR newborn rats is based on the normal birth weight. Those pups whose birth weight was below the 10th percentile of normal birth weight were defined as IUGR rats, and the litter size was culled to five pups per litter to assure adequate nutrition until weaning. The newborn pups from control group were considered as normal birth weight group “Control d1” and those newborn pups from the undernutrition group were labeled as IUGR group “IUGR d1”. Newborn IUGR rats continued to be reared by diet-restricted mothers that receive normal food intake through lactation, the control pups were reared by control mothers. Both group rats were raised until 6 weeks of age. Six-week control rats were labeled as “Control 6wks”, while 6-week IUGR rats were labeled an “IUGR 6wks” (Figure 1).

### Hypoxic pulmonary hypertension rat model

Hypoxic pulmonary hypertension model was established according to our previous experiments [30,31]. Briefly, 4-week IUGR rats and the age-matched control rats were placed in a normobaric hypoxic chamber with a fraction of inspiratory oxygen concentration of 12% for two weeks. During the experiment, the chamber was opened for 10 minutes every day for feeding and to change the animal cages. After a 2-week hypoxic intervention, rat pulmonary arterial pressure was measured, followed by lung tissue immunohistochemistry and isolation of pulmonary vascular endothelial cells. The control and IUGR rats with hypoxia treatment were labeled as “Control hypoxia” and “IUGR hypoxia”, respectively.

### Assessment of pulmonary arterial pressure

The right ventricular systolic pressure (RVSP) was used to reflect pulmonary arterial pressure. Measurement of RVSP of 6-week rats was performed according to our previous report [31]. Briefly, 6-week rats were anesthetized (Urethane, 50 mg/kg, intraperitoneal injection), followed by tracheal intubation and immediately connected to a ventilator with 12% O_2_ for the hypoxic group, and then the thorax was opened. A catheter connected to a pressure transducer was inserted into the right ventricle, and RVSP was recorded immediately. Normal saline was injected into the right ventricle through the needle at a pressure of 20 cmH_2_O at room temperature and the lung was flushed. Lungs were dissected once the effluent from the left atrium incision was clear of blood.

### Immunohistochemistry and physical measurements

Right lung tissues of different ages were removed, frozen in liquid nitrogen and stored at −80°C for Western blotting. Left lung tissues of different ages were isolated and perfused with ice cold 10% formalin at a pressure of 25 cm H_2_O and fixed for 24 hours at 4°C. The lung tissues were imbedded in paraffin, sectioned at 4–5 μm, and processed for light microscopic immunohistochemistry as the previously described [19,30]. Samples were incubated with a primary antibody against smooth muscle specific α-actin (α-SMA, clone: 1A4, Dako) overnight, followed by secondary goat antibody (HRP polymer) for 30 minutes at 37°C. At least eight slides from each lung were taken and then analyzed.

The percentage of pulmonary arterial medial thickness occupied by smooth muscle was determined according to our previous study [30]. Small pulmonary arteries of 50-150 μm in diameter were investigated via a systematic sampling method to evaluate random, non-overlapping calibrated fields for each variable. The terminal bronchioles were used as the independent landmark for selecting small pulmonary arteries [32]. We used Image Pro Plus software (Media Cybernetics) to make the measurements. Tissue slides were analyzed by technician who was without knowledge of the group from which the tissue was taken.

### Isolation of pulmonary vascular endothelial cells (PVEC)

Pulmonary vascular endothelial cells (PVEC) were isolated by magnetic-activated cell sorting (MACS) as described earlier [19]. Fresh rat lungs were sliced and then incubated with collagenase A. The suspensions were filtered, centrifuged, and washed three times in phosphate buffer saline (PBS) containing 0.5% bovine serum albumin (BSA) and 2 mM EDTA. The final pellets were resuspended in 100 μl of PBS with 0.5% BSA and 2 mM EDTA per 10^7^ total cells, and then incubated with PECAM-1 (555027, BD) for 15 minutes at 4°C. After washing, anti-PE MicroBeads (130-048-801, Miltenyi Biotec) were added to the cell suspension for 15 minutes at 4°C. The PVEC positively labeled with the magnetic microbeads linked to PECAM-1 antibody were selected with the separation unit, and kept at −80°C until use. The proportion of positive PVEC after MACS was about 92% [19], there were no significantly statistical difference between groups.

### Western blot detection for α-SMA and endothelin-1 proteins

Total protein extracts and protein concentrations were prepared according to our previous studies [19,30]. Thirty micrograms of protein extracts were resolved on 8-10% SDS polyacrylamide gels for α-SMA (42kDa) and ET-1 (21kDa) proteins. Proteins were transferred onto a polyvinylidene fluoride (PVDF) membrane using a BioRad gel blotting apparatus. Membranes were incubated with a primary antibody against α-SMA and ET-1 overnight at 4°C and with a peroxidase-conjugated secondary antibody for 60 minutes at room temperature. Peroxidase was visualized by enhanced chemiluminescence (ECL) and exposure to ECL films for appropriate times. The bands of Western blots were quantitated by densitometry and normalized with β-actin (42kDa) using Image Pro Plus software. Protein level is expressed as a percentage of normal control ± standard error of the mean (SEM).

### Quantitative real-time PCR for ET-1

Total RNA was isolated from PVECs according to the RNeasy protocol (Axygen). RNA was reverse-transcribed using a reverse transcriptase kit (TAKARA) according to manufacturer’s protocols. Real-time quantitative PCR was performed by the ABI Prism 7500 Instrument following the TAKARA protocol. β-actin was used as an internal control. Primers for ET-1 and β-actin are as follows: forward: 5’-aagcagacaaagaactccgag-3’, reverse: 5’-cgctttcaactttgcaactcg-3’; forward: 5’-gccaaccgtgaaaagatg-3’, reverse: 5’-tgccagtggtacgaccag-3’, respectively.

### Chromatin immunoprecipitation assay

Chromatin immunoprecipitation (ChIP) assay was performed as described in a previous study [19]. After the isolated PVEC were fixed and cross-linked, the cell pellets were suspended in cell lysis buffer and centrifuged, and the supernatants were discarded. The pellets were resuspended in SDS lysis buffer, sonicated, centrifuged and aliquots of soluble chromatin were collected. Aliquots of the supernatants were retained for representing the input chromatin. Aliquots were incubated overnight at 4°C with one of the following antibodies: 5 μl anti-acetylaled Histone H3 (06–599, Millipore), 5 μl anti-acetylated Histone H3 (Lys9/18) (H3K9/18, 07–593, Millipore), 5 μl anti-acetylaled Histone H4 (06–866, Millipore), anti-Hypoxia Inducible Factor 1 α (MAB5382, Millipore). The immune complexes were precipitated with Protein A beads and then eluted. The input, unbound and bound fractions were incubated with 5 M NaCl to reverse crosslink. The DNA fragments were purified using the Qiaquick PCR Purification kit (QIAGEN). The DNA fragments containing ET-1 site-specific sequences were quantified by real-time PCR. Relative quantification of PCR products were based on value differences between the bound and input using the △△C_t_ method. The PCR primers for the ET1 gene promoter were as follows: ET1 promoter A1, F5’-ttgcctgtgggtgactaatc-3’, R5’-ccttcaccggagcgaaag-3’ (−197 to +25); ET1 promoter A2, F5’-cctcttgattcttgaactctggg-3’, R5’-attagtcacccacaggcaac-3’ (−397 to −179).

### Statistic analysis

Values are expressed as mean ± SEM (standard error of mean). The continuous variables in the different groups were compared by one-way ANOVA. Multiple comparisons were performed utilizing the Student-Newman-Keuls test. A difference of *P <* 0.05 was considered to be statistically significant.

## Results

### Establishment of rat IUGR model

In the present study, the 10^th^ and 90^th^ percentiles for normal newborn rat birth weight were 5.87 g and 7.60 g, respectively. Newborn rats weighing less than 5.80 g were considered to be IUGR. The average birth weight of normal newborn rats (n = 276) was 6.80 g ± 0.044 g, while the average birth weight of newborn rats from the diet restricted group (n = 305) was 5.10 g ± 0.037 g. There was statistically significant difference between the two groups (*P* < 0.001). The sampling age of different experimental group rats were seen in Figure 1.

### Right ventricular systolic pressure (RVSP) of 6-week, hypoxia-treated rats

**Figure 2:**
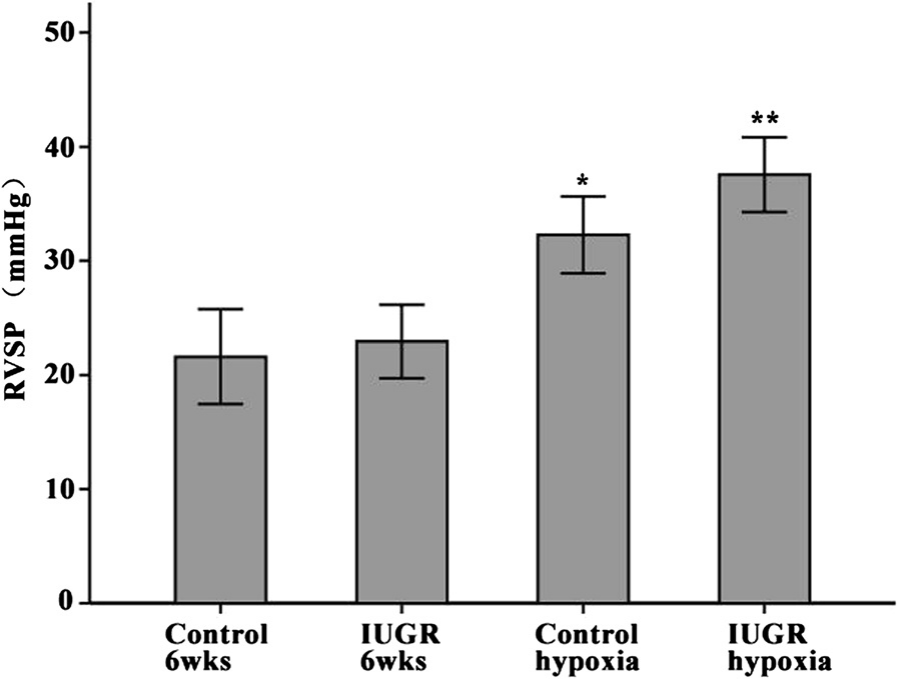
**Comparison of right ventricular systolic pressure (RVSP) of different groups.** *P < 0.05 as compared with Control 6wks (weeks), and IUGR 6wks, respectively; **P < 0.05 as compared with Control 6wks, IUGR 6wks, and Control hypoxia, respectively. (n ≥ 8).

RVSP of 6-week control rats was 21.6 ± 2.08 mmHg, and the RVSP of 6-week IUGR rats was 22.93 ± 1.60 mmHg, there was no significant statistical difference between the two groups (*P* = 0.622, Figure 2). After hypoxia intervention, the RVSP of both groups increased significantly. The RVSP of the IUGR hypoxia group was statistically significantly higher than that of the Control hypoxia group (*P* = 0.04), which indicates that IUGR rats were more sensitive to hypoxia than normal control rats.

### Pulmonary histological changes in 1-day, 6-week, hypoxia-treated rats

The percentage of medial thickness in small pulmonary arteries of 50–150 μm diameter was analyzed. The medial/(medial + lumen) thickness ratios of IUGR d1 group rats were lower than those of Control d1 group rats, but there was no statistically significant difference between the two groups (0.2678 ± 0.012 versus 0.2384 ± 0.013, *P* = 0.152). Although the medial thickness ratios of IUGR 6wks group rats were slightly higher than those of age-matched control rats, there was no statistically significant difference between the two groups (0.3397 ± 0.011 versus 0.3393 ± 0.015, *P* = 0.986).

**Figure 3:**
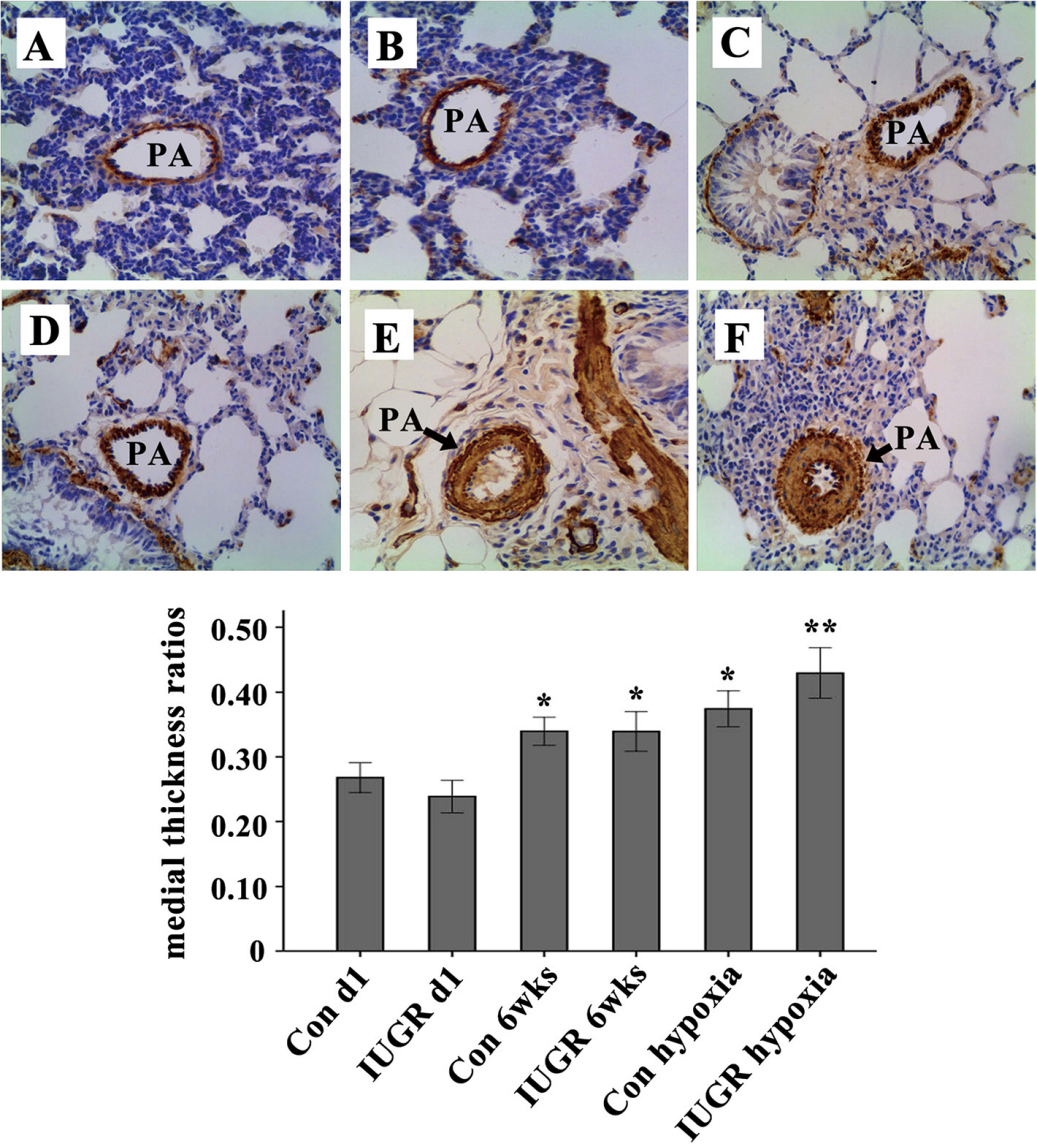
**Immunohistochemical staining for a-SMA in lung tissue sections.** Representative lung histological sections of Control d1 (**A**), IUGR d1 (**B**), Control 6wks (**C**), IUGR 6wks (**D**), Control hypoxia (**E**), and IUGR hypoxia (**F**) are shown demonstrating hypertrophy of the arterial medial wall induced by hypoxia. Note significant medial thickness (**E** and **F**), especially in IUGR hypoxia group (x 200). The bar graph represents the medial/(medial + lumen) thickness ratios of the small pulmonary arteries in various group rats, *P < 0.05 as compared with control d1, and IUGR d1, respectively; ^**^P < 0.05 as compared with control d1, IUGR d1, Control 6wks, IUGR 6wks, and Control hypoxia, respectively. PA = pulmonary artery. (n = 10).

Significant histological changes compatible with pulmonary vascular remodeling were observed in hypoxia-treated rats, especially in IUGR rats. The medial thickness ratios of hypoxia-treated rats were significantly higher than those of age-matched control and IUGR rats. Moreover, the medial thickness ratios of IUGR hypoxia group rats were higher than those of Control hypoxia group (0.4295 ± 0.019 versus 0.3743 ± 0.014). There was a statistically significant difference between the two groups (*P* = 0.019) (Figure 3).

### Lung tissue α-SMA levels

**Figure 4:**
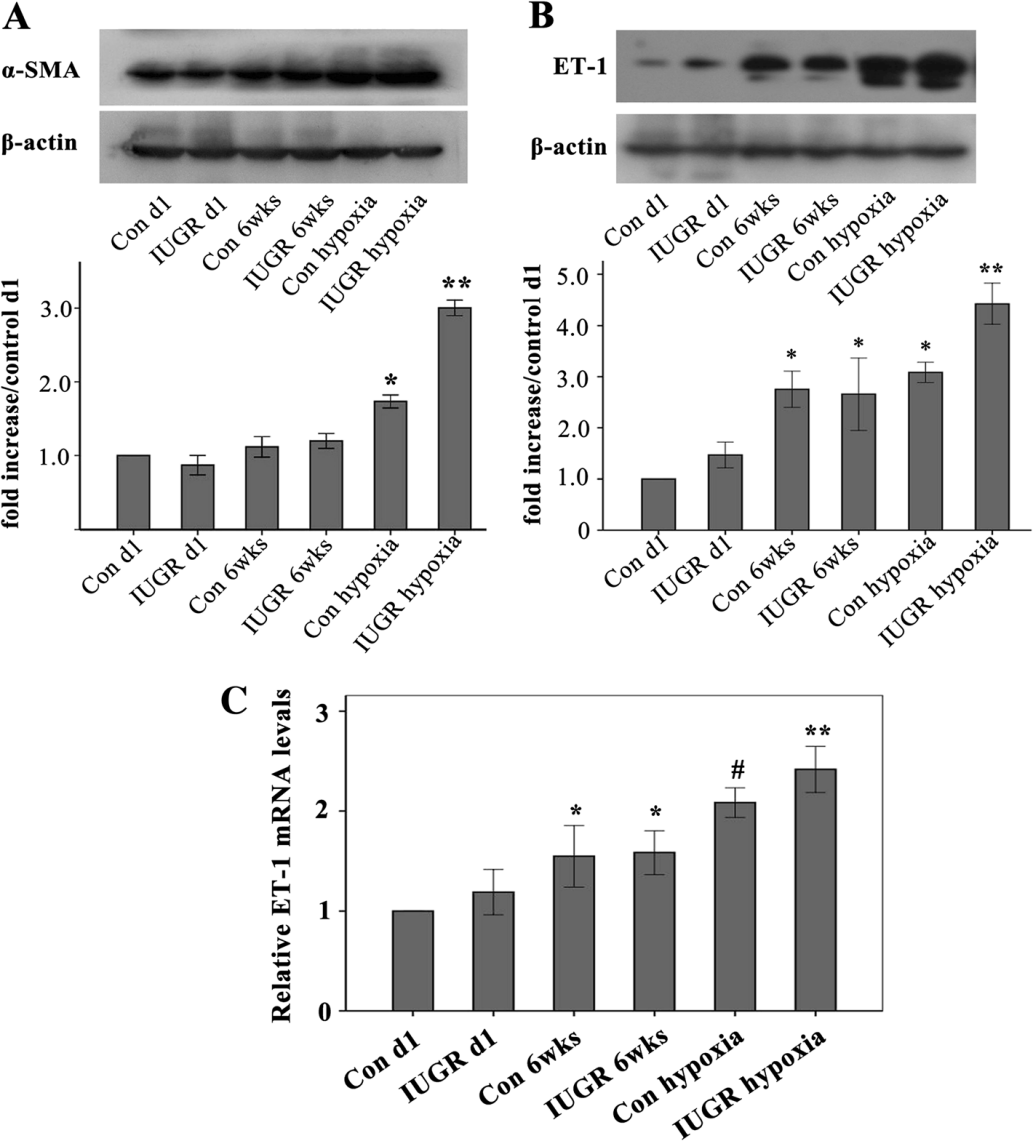
**Expression of α-SMA and ET-1 proteins was measured using Western blot analysis.** Lung tissue protein extractions were used for α-SMA expression analysis (**A**). The bar graph represents the α-SMA protein relative levels (percent of Control d1 ± SEM). *P < 0.05 as compared with Control d1, IUGR d1, Control 6wks, and IUGR 6wks groups, respectively; **P < 0.05 as compared with Control d1, IUGR d1, Control 6wks, IUGR 6wks, and Control hypoxia groups, respectively (**A**); note increased α-SMA expression in the IUGR hypoxia group. PVEC protein extractions were used for ET-1 expression analysis (**B**). The bar graph represents the ET-1 protein relative levels (percent of control d1 ± SEM); *P < 0.05 as compared with Control d1 and IUGR d1; **P < 0.05 as compared with Control d1, IUGR d1, Control 6wks, IUGR 6wks, and Control hypoxia groups, respectively (**B**); note increased ET-1 expression in the IUGR hypoxia group. β-actin protein expression serves as an internal control and is used to normalize the protein band intensity (n = 4). The relative ET-1 mRNA levels were examined by quantitative real-time PCR (**C**), *P < 0.05 as compared with control d1, and IUGR d1, respectively; ^#^*P* < 0.05 as compared with control d1, IUGR d1, Control 6wks, IUGR 6wks, and IUGR hypoxia, respectively; ^**^P < 0.05 as compared with control d1, IUGR d1, Control 6wks, IUGR 6wks, and Control hypoxia, respectively (n = 4). Con = Control, SEM = standard error of mean.

Lung tissue α-SMA protein expression levels reflecting pulmonary vascular remodeling in IUGR hypoxia and Control hypoxia groups were significantly increased (Figure 4A). Furthermore, the α-SMA level of the IUGR hypoxia group was higher than that of Control hypoxia group, which yielded a significant difference (*P* < 0.01). This further indicated that the sensitivity to hypoxia in IUGR rats was significantly higher than the normal control group.

### Endothelin −1 (ET-1) expression of PVEC

Although the ET-1 protein level of the IUGR d1 group was higher than the age-matched control group, there was no significantly statistical significance (*P* > 0.05, Figure 4B). The ET-1 protein level of the IUGR 6wks group was similar to that of the Control 6wks group, there was no significant difference (*P* > 0.05). However, the ET-1 expression level in the IUGR hypoxia group was significantly higher than that in Control hypoxia group, and there was a statistically significant difference between the two groups (*P* = 0.02). Similar to protein expression pattern, the ET-1 mRNA expression showed a similar trend (Figure 4C). These results indicate that the increased ET-1 level of IUGR hypoxia group rats might be responsible for the increased RVSP and pulmonary vascular remodeling changes.

### The ET-1 gene promoter histone code of PVEC

**Figure 5:**
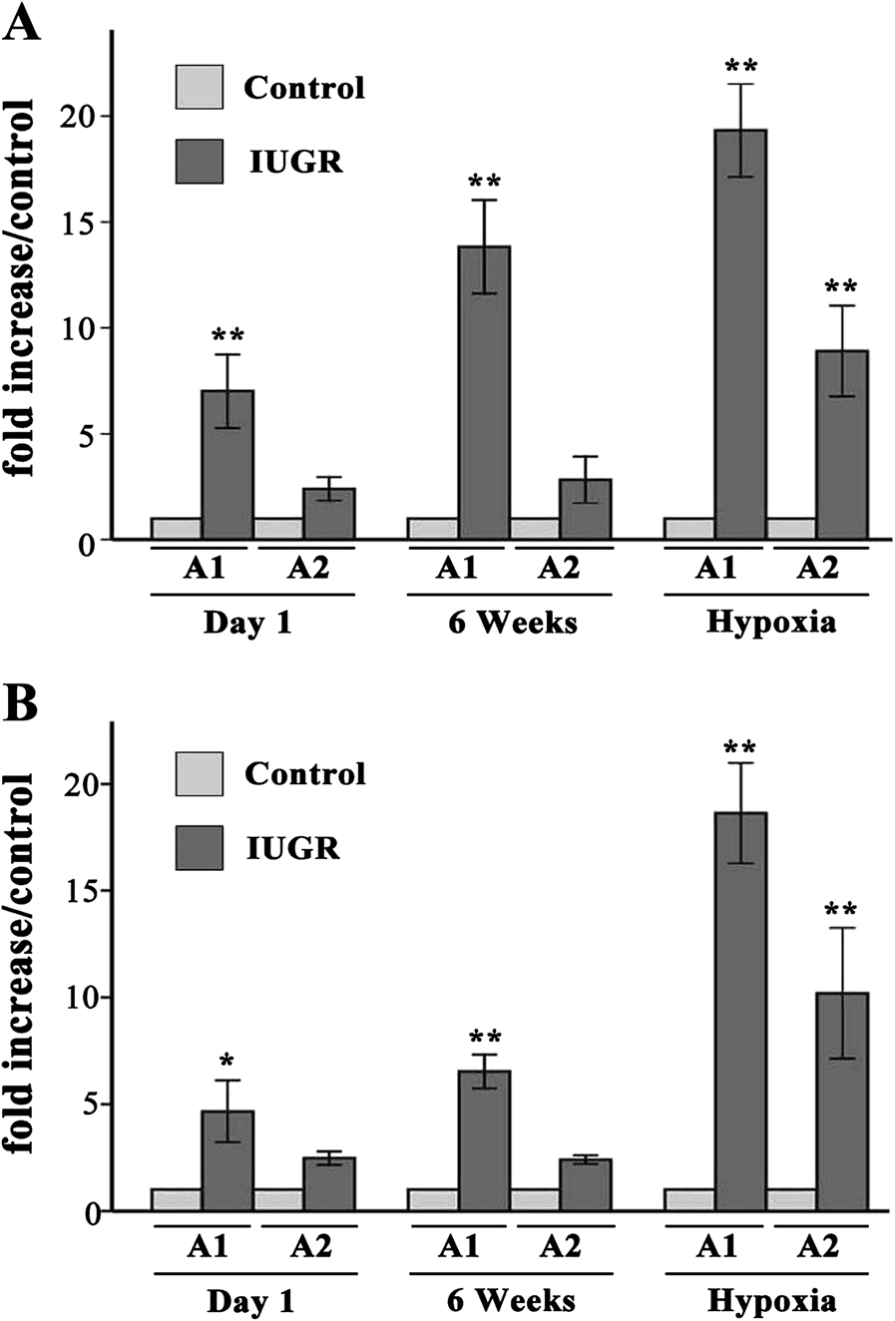
**Comparison of histone H3 (A) and H3K9/18 (B) acetylation levels at the rat ET-1 promoter between IUGR and the control using ChIP and relative quantitative real-time PCR.** A1 and A2 represent two areas of the ET-1 gene promoter (−197 to +25 and −397 to −179), respectively. Data are expressed as IUGR percent of the Control ± SEM. *P < 0.05 and **P < 0.01 as compared with Control groups, respectively.

Two sites along the ET-1 promoter were analyzed for acetylated histone H3 and H4. Acetylation levels of every specific site in IUGR rats were quantified by ChIP/real-time PCR and expressed as an increased fold relative to the age-matched controls. The levels of acetylated histone H3 in the ET-1 promoter A1 region of PVEC from IUGR d1, IUGR 6wks, and IUGR hypoxia groups were significantly higher than those from the age-matched control groups (*P* < 0.01, Figure 5A). While in the ET-1 promoter A2 region, only the IUGR hypoxia group was higher than Control hypoxia group (*P* = 0.021). The trend of acetylated H3K9/18 levels in the ET-1 promoter of PVEC was similar to the acetylated histone H3 change (Figure 5B).

**Figure 6:**
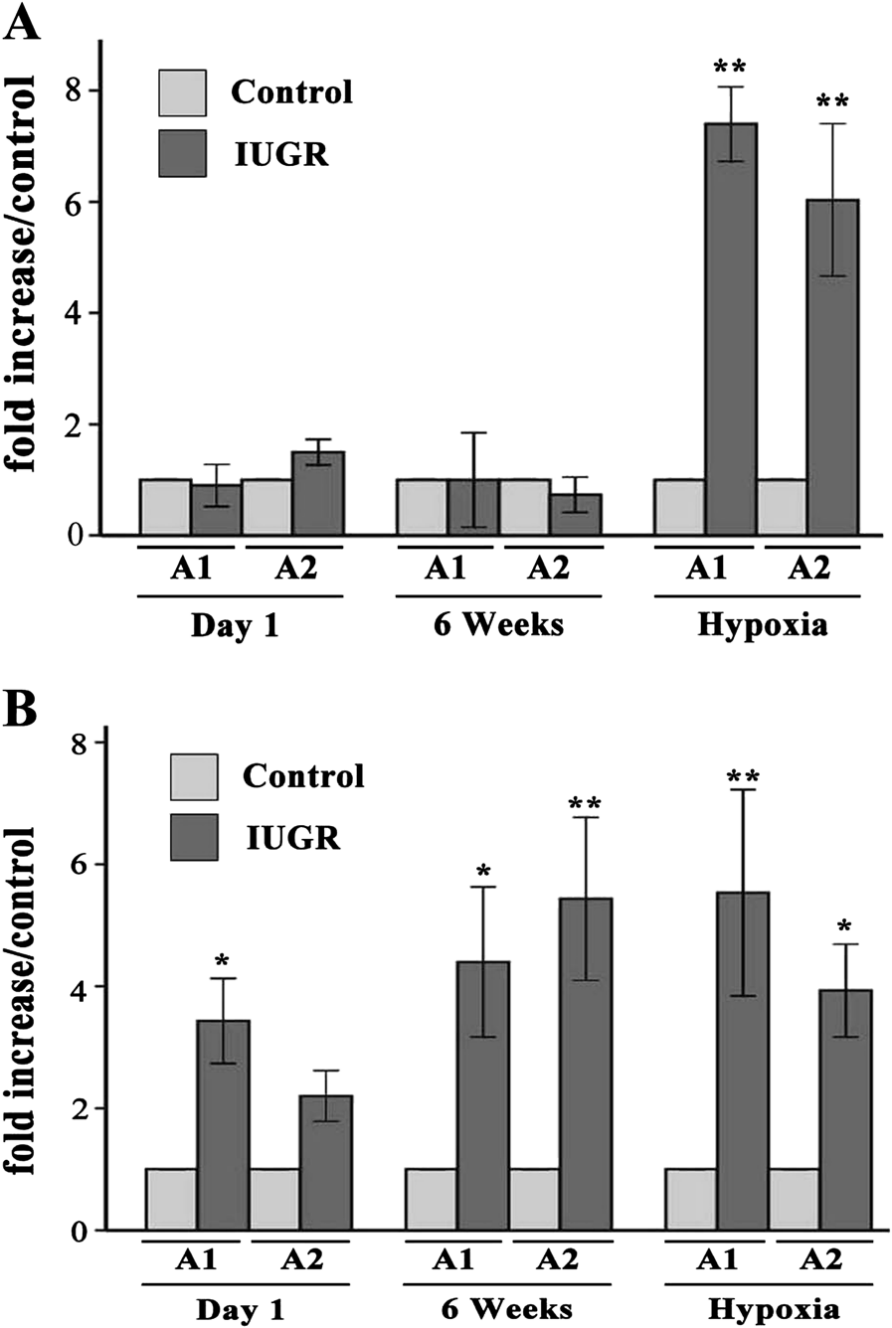
**Comparison of histone H4 acetylation (A) and HIF-1α binding (B) levels at the rat ET-1 promoter between IUGR and the control using ChIP and relative quantitative real-time PCR.** A1 and A2 represent two areas of the ET-1 gene promoter (−197 to +25 and −397 to −179), respectively. Data are expressed as IUGR percent of the Control ± SEM. *P < 0.05 and **P < 0.01 as compared with Control groups, respectively.

The levels of acetylated histone H4 in the ET-1 promoter regions from IUGR d1 and 6wks groups were similar to the age-matched control groups (Figure 6A). However, the level of acetylated histone H4 in the ET-1 promoter A1 and A2 regions of the IUGR hypoxia group was significantly higher than that of Control hypoxia group, there were statistically significant differences (*P* = 0.011 and *P* = 0.021, respectively).

### Hypoxia inducible factor-1α (HIF-1α) enrichment in ET-1 gene promoter of PVEC

To explore the role of transcription factor HIF-1α in hypoxia-induced pulmonary hypertension, HIF-1α binding levels of the ET-1 gene promoter in PVEC between the IUGR and control groups were analyzed. The HIF-1α binding levels in the ET-1 gene promoter A1 region from the IUGR d1, 6wks and hypoxia groups were higher than the age-matched control groups, and there were statistically significantly statistical differences (*P* < 0.05, Figure 6B). There were also similar changes in HIF-1α binding levels of the ET-1 gene promoter A2 region, except between the IUGR d1 and Control d1 groups.

The ChIP experiments revealed that histone acetylation modification of the ET-1 gene promoter in IUGR rats might induce transcription factor HIF-1α accumulation in the ET-1 promoter region, which would lead to the ET-1 gene transcription and protein expression.

## Discussion

The ‘histone code’ hypothesis proposes that covalent modifications of the histone proteins contribute to transcriptional regulation. These histone modifications provide a mechanism that allows encoding information of successive generations of cell division [33,34]. Generally, increased levels of histone acetylation are associated with increased transcriptional activity, whereas decreased levels of acetylation are correlated with suppressed gene expression. Our data demonstrate that the open chromatin domains marked by histone H3 and H3K9/18 acetylation at the proximal promoter of ET-1 in IUGR rats are essential for transcription. Upregulated ET-1 protein expression in PVEC from IUGR hypoxia rats is closely associated with the presence of increased acetylated H3 histones. Reduction of these markers in Control hypoxia rats results in the decreased ET-1 protein expression.

In the present study, PVEC isolated from IUGR day1 rats shows a significant increase in H3 and H3K9/18 acetylation at the proximal promoter of ET-1, and can be continued until 6 weeks after birth. These epigenetic markers would contribute to the higher sensitivity in IUGR rats in later life to hypoxia, leading to more pronounced PAH or pulmonary vascular remodeling than the normal rats. The increased histone acetylation of the ET-1 gene in the IUGR hypoxia rats up-regulated ET-1 protein expression, inducing a more significant PAH. In general, histone acetylation will lead to increase in gene expression. However, under normal oxygen conditions, although ET-1 protein expression in IUGR newborn rats was higher than age-matched control group, there was no significant difference between the two groups. Moreover, ET-1 protein level in 6-week IUGR rats was similar to the age-matched control group. The ET-1 protein expression mode in part explained the fact that 6-week IUGR rats, under normoxia, did not produce more significant pulmonary arterial pressure and pulmonary vascular remodeling than the age-matched control rats. Our data demonstrates that the histone acetylation of the ET-1 gene in 6-week IUGR rats is not enough to cause ET-1 protein expression. In addition to histone acetylation, other epigenetic alterations including RNA interference might also be involved in regulation of the ET-1 gene to suppress expression of ET-1 protein. While these possible epigenetic modifications would be likely to induce ET-1 protein expression only in specific stress situations. However, this balance was broken by hypoxia intervention. The ET-1 protein level of PVEC from the IUGR hypoxia group was significantly higher than Control hypoxia group. The up-regulated ET-1 protein level was consistent with increased RVSP and pulmonary vascular remodeling observed in IUGR hypoxia rats. The molecular mechanism responsible for the increased ET-1 protein expression in IUGR hypoxia rats is likely to be strongly related to the recruitment of hypoxia-induced transcriptional factors in the activated (increased histone acetylation) ET-1 promoter, especially the recruitment of HIF-1α.

**Figure 7:**
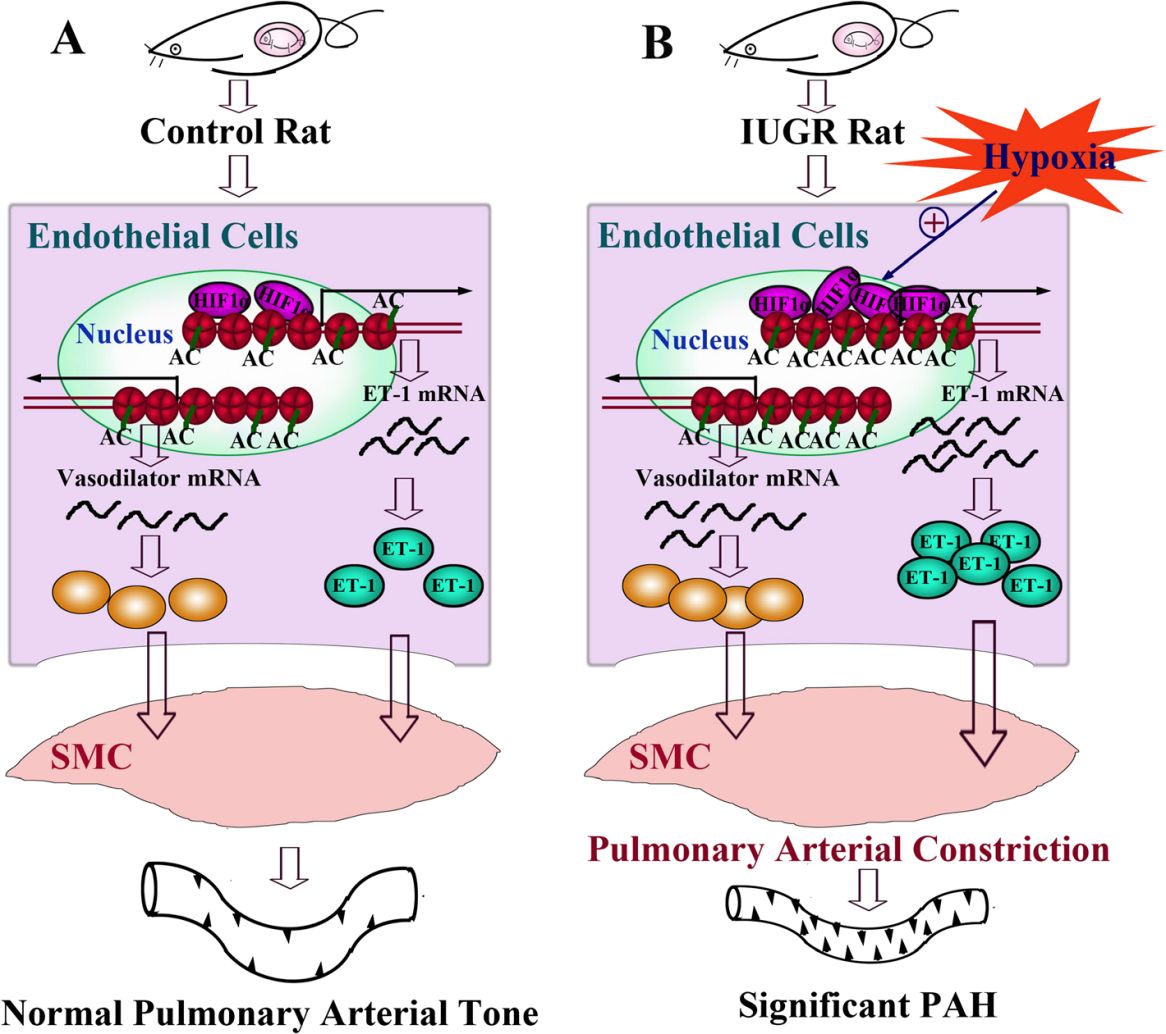
**Development of hypoxic pulmonary arterial hypertension following IUGR.** Maternal nutrient restriction increased the histone acetylation and transcription factor HIF-1α binding levels in the ET-1 gene promoter of pulmonary vascular endothelial cells (PVEC) in IUGR rats. These epigenetic changes would induce IUGR individual in later life to be highly sensitive to hypoxia, resulting in more significant pulmonary hypertension (**B**). AC = (Histone) acetylation, SMC = smooth muscle cells, PAH = pulmonary arterial hypertension.

HIF-1 is a heterodimer consisting of HIF-1α and HIF-1β subunits, which functions as a key regulator responsible for hypoxia-inducible gene expression [35,36]. In normoxia, the HIF-1α proteins are rapidly degraded. While during hypoxia, HIF-1α becomes stabilized and translocates to the nucleus, where it forms the activated HIF complex and co-induces target gene expression. Our study revealed that the recruitment of HIF-1α in the ET-1 promoter in PVEC of IUGR newborn rats was significantly increased, and continued until 6 weeks after birth. Furthermore, this trend persisted even after hypoxia intervention. The increased HIF-1α binding level in the ET-1 promoter of IUGR rats would contribute to ET-1 protein expression, which further exacerbates pulmonary arterial pressure and pulmonary vascular remodeling in IUGR rats (Figure 7).

A previous report demonstrated that in mice undernutrition during gestation would induce pulmonary vascular dysfunction in the offspring by a DNA methylation of lung tissue [18]. Different from their study at a tissue level, we focused on a cellular level to investigate the role of epigenetics of the special gene in development of hypoxic PAH following IUGR. Whether RNA interference, DNA methylation, or other hypoxia-induced transcription factors were involved in development of hypoxic PAH following IUGR, whether these epigenetic changes were maintained into next generation or could be reversed, would deserve further investigation.

## Conclusions

In summary, we found that hypoxia induced IUGR rats experience significant PAH and pulmonary vascular remodeling. The response characteristics of IUGR to hypoxia were strongly correlated with the increased histone acetylation and HIF-1α binding levels in the ET-1 gene core promoter region. These findings suggest that epigenetics are closely associated with the development of hypoxic PAH following IUGR, further providing a new insight for improved prevention of IUGR-related PAH.

## Competing interests

The authors declare that they have no competing interests.

## Authors’ contributions

XFX carried out the experimental models and performed the statistical analysis. YL carried out the epigenetic experiments. WZG carried out the immunostaining. LLT and JKW participated in carrying out the experimental models. LYZ designed the experimental models. XFX and LZD conceived the study, and participated in its design and coordination, and prepared the manuscript. All authors read and approved the final manuscript.
